# Insects and Disease

**Published:** 1905-09-09

**Authors:** 


### The Hospital.

A Journal of

The Medical Sciences and Hospital Administration.

Vol. XXXVIII.—No. 989. SATURDAY, SEPTEMBER 9, 1905.

### Insects and Disease.

We have somewhere seen it stated that, shortly after the identification of members of the genus *Anopheles* as the hosts and carriers of the parasite which produces intermittent fever when communicated to mankind, and in view of the extended scientific investigations thence arising, the leaders of one of the Anti-Vivisection Societies issued to their supporters a pamphlet for private circulation, advocating the immediate formation of a special branch of the Society, for the protection of what was described as "the gentle and intelligent mosquito" against the cruelties said to be practised upon it by iniquitous persons professedly engaged in the pursuit of that knowledge which the members of the Society neither possessed nor desired, and which, like the Chinese navigator when he first saw a sextant, they pronounced to be "utterly barbarian and altogether useless." It was declared that even the salivary ducts of the tortured insects were not held sacred by the vivisectors, and that humanity cried out aloud for active measures of protection. We are not quite sure of the extent to which this interesting development of the work of the Society has yet been carried, or of the success which has attended upon the "mosquito branch" of its operations; but the proceedings in South Africa of the British Association for the Advancement of Science have rendered it manifest that these operations cannot for the future be confined to mosquitoes, and that the sufferings of many varieties of flies and ticks must also be taken into account. Mr. A. E. Shipley, F.R.S., has been reading at Johannesburg a paper on the "Infinite Torment of Flies," in which he has brought together, into one appalling picture, the influence exerted by these small creatures in the dissemination of disease, a picture which might even excite, in the ill-regulated minds of persons capable of condoning experiment, a desire for the complete extermination of some of the most graceful and most beautiful of the winged portion of creation. Mr. Shipley told his hearers that the history of the late war in South Africa definitely showed that common house flies played a large part in carrying the bacilli of enteric fever from sources of infection to the food of man, and in thus spreading the disease. He considered it possible that the same insects might convey the bacilli of anthrax to wounded surfaces, and also that flies, ants, and other similar creatures, were capable of disseminating the plague bacillus from man to man.

Passing on, however, from what may be called the mischief accidentally wrought by insects to that which seems to be inseparable from the scheme of their existence, Mr. Shipley explained the totally new light in which, during the last few years, it has become necessary to regard them. After a glance at the *culicidæ* as the carriers of the blood parasite, *Filaria nocturna*, he gave a brief history of the *Anopheles* in its relation to intermittent, and of the *Stegomyia* in its relation to yellow fever, pointing out how much the difficulty of protection against the latter was increased by its diurnal habits, and how necessary it therefore was to pursue active measures for the destruction of the larvæ. He passed on to the tse-tse fly, *Glossina morsitans*, which communicates fatal diseases to both men and animals over extensive districts of South Africa, the so-called "fly-belts," and to the still more dangerous *Glossina palpalis*, the carrier of the trypanosoma which occasions sleeping sickness, a disease by which the inhabitants of the single island of Buvuma have lately been reduced from 22,000 to 8,000, and by which whole districts in the interior of Africa have been depopulated. It was first noticed in Uganda in 1901, and by the middle of 1904 100,000 people had been killed there by it. Finally, he referred to a group of diseases the study of which is only commencing, but which affect dogs and cattle in various parts of the world, and are known as Texas fever, tick fever, heart water, and under many other French, German, Italian, and Spanish names. These are found to be caused by various species of a Protozoon named *Piroplasma*, and may be collectively described as Piroplasmosis. Under the names of Rocky Mountain fever, spotted fever, or tick fever, they also attack mankind throughout the western half of the United States, and it is known that they are communicated from one subject to another by the bites of mites or ticks. The protozoa concerned are chiefly found within the red corpuscles; but many points in their natural history have still to be investigated.

The conception of insect life as the immediate cause, or channel of diffusion, of a large number of the most fatal of the diseases which affect either mankind or animals, and the consequent disappearance of any belief in the hurtful influence of "climate," is undoubtedly one of the most important of the scientific acquisitions of the last few years, and is likely to be one of the most far-reaching in its effects. By the destruction of larvæ, and by the use of mosquito nets, yellow fever has already been banished from Havana, and ague almost entirely from Ismailia and from the Straits Settlements. Further progress will no doubt be made in the same direction; but, in the meanwhile, one of the most curious facts in the natural history of the diseases concerned is that, in some of them, the insect carriers appear to be merely passive, to receive the parasite from an infected subject and to pass it on to a healthy one, while in others, as in ague, the temporary host appears to play an essential part in the development of the amœba. The difference seems to point to different requirements in the direction of preventive treatment; to the extermination of larvæ in the one case, and possibly to that of infected herds or animals in the other.

